# On the Efficacy and Mediation of a One-on-One HIV Risk-Reduction Intervention for African American Men Who Have Sex with Men: A Randomized Controlled Trial

**DOI:** 10.1007/s10461-014-0961-2

**Published:** 2014-12-02

**Authors:** John B. Jemmott III, Loretta Sweet Jemmott, Ann O’Leary, Larry D. Icard, Scott E. Rutledge, Robin Stevens, Janet Hsu, Alisa J. Stephens

**Affiliations:** 1 Annenberg School for Communication and Department of Psychiatry, Perelman School of Medicine, University of Pennsylvania, 3535 Market Street, Suite 520, Philadelphia, PA 19104-3309 USA; 2 School of Nursing, University of Pennsylvania, Philadelphia, PA USA; 3 Division of HIV/AIDS Prevention, Centers for Disease Control and Prevention, Atlanta, GA USA; 4 School of Social Work, Temple University, Philadelphia, PA USA; 5 Department of Psychiatry, Perelman School of Medicine, University of Pennsylvania, Philadelphia, PA USA; 6 Center for Clinical Epidemiology and Biostatistics, Perelman School of Medicine, University of Pennsylvania, Philadelphia, PA USA

**Keywords:** HIV, Men who have sex with men, African Americans, Intervention, Sexual behavior

## Abstract

We examined the efficacy and mediation of *Being Responsible for Ourselves* (BRO), an HIV/STI risk-reduction intervention for African American men who have sex with men (MSM), the population with the highest HIV-diagnosis rate in the US. We randomized African American MSM to one of two interventions: BRO HIV/STI risk-reduction, targeting condom use; or attention-matched control, targeting physical activity and healthy diet. The interventions were based on social cognitive theory, the reasoned-action approach, and qualitative research. Men reporting anal intercourse with other men in the past 90 days were eligible and completed pre-intervention, immediately post-intervention, and 6 and 12 months post-intervention surveys. Of 595 participants, 503 (85 %) completed the 12-month follow-up. Generalized-estimating-equations analysis indicated that, compared with the attention-matched control intervention, the BRO intervention did not increase consistent condom use averaged over the 6- and 12-month follow-ups, which was the primary outcome. Although BRO did not affect the proportion of condom-protected intercourse acts, unprotected sexual intercourse, multiple partners, or insertive anal intercourse, it did reduce receptive anal intercourse compared with the control, a behavior linked to incident HIV infection. Mediation analysis using the product-of-coefficients approach revealed that although BRO increased seven of nine theoretical constructs it was designed to affect, it increased only one of three theoretical constructs that predicted consistent condom use: condom-use impulse-control self-efficacy. Thus, BRO indirectly increased consistent condom use through condom-use impulse-control self-efficacy. In conclusion, although BRO increased several theoretical constructs, most of those constructs did not predict consistent condom use; hence, the intervention did not increase it. Theoretical constructs that interventions should target to increase African American MSM’s condom use are discussed.

## Introduction

The rate of HIV diagnosis among African American men who have sex with men (MSM) is alarming. Although African Americans represent only 13 % of the US population, 46 % of HIV diagnoses from 2008 through 2011 were among African Americans, and MSM accounted for the largest number of African Americans living with HIV/AIDS [1]. In 2011, the estimated rate of HIV diagnosis was 112.8 per 100,000 in African American men compared with 14.5 in white men [1], and there were more estimated HIV diagnoses in the male-to-male contact transmission category in African American men (11,805) than in white men (10,375).

Despite the high rate of HIV diagnosis among African American MSM, limited progress has been made in developing efficacious interventions for this population. A systematic review of behavioral interventions for MSM published between 1988 and 2010 [2] identified 33 studies, of which nine (27 %) met the criteria for evidence-based interventions, but only one focused on African American MSM. That randomized controlled trial (RCT) tested a group intervention that was implemented using a weekend retreat [3]. Averaged over 3- and 6-month post-intervention assessments, participants in the intervention were less likely to report unprotected insertive anal intercourse with casual male partners than were those in a wait-list comparison condition.

Other recent RCTs of HIV risk-reduction interventions for African American MSM have suffered from small samples, short follow-up periods, or nonsignificant results. A RCT testing an HIV risk-reduction intervention for African American MSM utilizing group sessions found no difference between intervention and control conditions on sexual-risk behavior 3 months post-intervention [4]. A pilot study testing a small-group intervention for HIV-positive African American men aged 50 and older also found no difference in condom use between the HIV/STI risk-reduction intervention and the control group 3 months post-intervention [5].

The interpretation of the results of RCTs of HIV risk-reduction interventions can be improved through the use of mediation analysis [6, 7], which helps identify the aspects of an intervention responsible for its effect or lack of effect. Although several mediation analyses of HIV risk-reduction interventions have been published [8–12], none focused on African American MSM. Accordingly, the mechanisms responsible for efficacy in successful interventions [3] and those responsible for lack of efficacy in unsuccessful interventions [4, 5, 13] are largely unknown. Thus, despite 30 years of research on HIV prevention, we still do not understand the mechanisms that underlie behavior change in African American MSM, the population at highest risk for HIV in the US.

Here we report the efficacy and mediation of an intervention to increase consistent condom use in African American MSM. The intervention, developed based on the social cognitive theory [14] and the reasoned action approach [15, 16] integrated with extensive formative research with the target population [17] to ensure that it was culturally congruent, was tested using a RCT design. A one-on-one intervention was employed to allay the fear of those MSM concerned about revealing their sexual involvement with men by virtue of participating in a group intervention, a fear that may be particularly concerning in African American MSM [18, 19]. One-on-one HIV risk-reduction interventions have been efficacious in several RCTs, reducing self-reported sexual-risk behaviors and biologically confirmed STIs [20–22]. Participants were randomized to *Being Responsible for Ourselves* (BRO), a three-session one-on-one HIV/STI risk-reduction intervention designed to increase consistent condom use or a three-session one-on-one health-promotion intervention, which served as the attention-matched control group. We hypothesized that compared with the attention-control group, controlling for baseline consistent condom use, the HIV/STI risk-reduction intervention would increase consistent condom use during the 12-month post-intervention period, which was the primary outcome. To better understand our results, we also report a mediation analysis on theoretical constructs hypothesized to predict consistent condom use.

## Methods

Institutional review boards (IRB) at the University of Pennsylvania and Temple University approved this study. Because the Centers for Disease Control and Prevention (CDC) co-author was not engaged in direct contact with the participants, the CDC deferred approval to the IRB at the University of Pennsylvania. The study included African American MSM irrespective of their HIV status and sexual orientation self-identification. Potential participants were screened for eligibility. Men were eligible to participate if they were at least 18 years of age, self-identified as black or African American, were born a male, and reported having anal intercourse with a man in the previous 90 days.

Men were excluded if they reported having anal intercourse with only one main male partner in the past 90 days or had participated in an HIV/STI risk-reduction intervention in the past 12 months. Those eligible were invited to participate in *Project Being Responsible for Ourselves* (BRO) designed to reduce the chances that men develop devastating health problems, including cardiovascular diseases, cancers, and STIs, including HIV. Informed consent while blind to group assignment was required for participation. Participants were recruited in the Philadelphia area (a) through advertising in local newspapers read by African American MSM, (b) through community-based organizations (CBOs) serving African American MSM, (c) through recruitment flyers posted at colleges, universities, parks, bars, and adult bookstores, (d) through face-to-face recruitment at social events, activities, and parties where a large turnout of African American MSM was expected, and (e) through the referrals of participants (i.e., snowballing).

In a RCT design, computer-generated random number sequences were used to randomly assign participants to the BRO HIV/STI risk-reduction intervention or the attention-matched health-promotion intervention that served as the control condition using concealment of allocation techniques designed to minimize bias in assignment. The biostatistician generated the random assignments; the project director implemented the assignments. Participants were enrolled between April 2008 and March 2011, with all data collection completed by May 2012.

Participants who completed the pre-intervention questionnaire and attended Intervention Session 1, which occurred at the same visit, were enrolled in the trial. After enrollment, data collectors, but not intervention facilitators or participants, were blind to group assignments. We held the intervention and data-collection sessions at a university research center. Participants were compensated with $25 for the pre-intervention assessment, $25 for each of the three intervention sessions, $25 for the immediate post-intervention assessment, and $50 for each of the two follow-up assessments.

### Interventions

The HIV/STI risk-reduction and health-promotion interventions were developed based on social cognitive theory [14] and the reasoned action approach [15, 16], integrated with extensive formative research [17], including focus groups and pilot testing. Social cognitive theory has been used to develop interventions to change sexual-risk behaviors [20, 23–26] and a host of other health behaviors [27–31]. Most relevant here are the social-cognitive-theory constructs of “outcome expectancy,” beliefs about the consequences of a specific behavior, and “self-efficacy,” people’s confidence that they can execute a specific behavior; its emphasis on behavioral skills; and its methods for increasing skills, particularly practice with performance feedback (e.g., role-playing). The reasoned action approach is an extension of the theory of planned behavior [32], which itself is an extension of the theory of reasoned action [33]. Most relevant here are the reasoned action approach’s emphasis on the importance of salient beliefs, its notion that such beliefs may vary from population to population and from behavior to behavior, and its methods to identify such population-specific beliefs: namely, the use of qualitative research, including focus groups. Identifying the salient beliefs in the population and then employing intervention activities designed to influence those population-specific beliefs can result in an intervention that is both theoretically grounded and tailored to the population.

Consistent with the reasoned action approach, we conducted qualitative research, seven focus groups with African American MSM and one with representatives of CBOs that serve African American MSM, to ensure the intervention was tailored to the population. In addition, we conducted three pilot tests of the interventions. Each intervention consisted of three 90-min one-on-one individual sessions implemented during 3 consecutive weeks by specially trained facilitators using standardized intervention manuals. We utilized one-on-one interventions to allay concerns some African American MSM might have about revealing their sexual behavior with other men by virtue of participating in a group-based intervention [18, 19], a concern expressed in the focus groups with African American MSM and with representatives of CBOs serving African American MSM. Sessions 1 and 2 in each intervention included take-home assignments that the participants reviewed at the subsequent session. The delivery of each intervention was tailored to the information that the participants provided during the sessions, including information about behaviors relevant to the particular intervention, the context in which the behaviors occurred, and participants’ motivation for the behaviors.

The BRO HIV/STI risk-reduction intervention was designed to strengthen outcome expectancies expressed in focus groups with African American MSM, outcome expectancies that have been observed in other populuations, including the hedonistic outcome expectancy that using condoms would not interfere with sexual enjoyment [9, 11, 23, 24, 34–37], the prevention outcome expectancy that using condoms prevents STIs, including HIV [24, 38], and the self-evaluative outcome expectancy that using condoms would make the man feel good about himself [9, 23, 35]. The intervention was designed to address aspects of self-efficacy identified in the focus groups, including technical-skill self-efficacy to use condoms correctly without interfering with sexual enjoyment [8, 36, 37], impulse-control self-efficacy to exercise the necessary control to use condoms even when sexually excited, under the influence of alcohol or drugs, or in the presence of other triggers for unsafe sex [11, 24, 37], and skills and self-efficacy to negotiate condom use with sexual partners [8, 36, 37]. In addition, it was designed to increase knowledge regarding the risk of acquiring or transmitting HIV and other STIs, and perceived vulnerability to HIV infection or re-infection with a different strain of HIV.

In Session 1, a general introduction was designed to provide an overview, create enthusiasm, build trust, and help the facilitator learn about the participant as an individual, including his goals, reasons for participating, and sexual orientation self-identification. In the “Where Do You Draw the Line” activity, participants discussed their sexual-risk behavior, including when and where they had sex, the types of sex in which they engaged, and when they used and did not use condoms, information used later to tailor activities (e.g., role-plays) to the participants. Participants completed a risk-assessment activity on risky sexual practices, an activity that focus groups said would help men to recognize their personal risk of HIV, an activity also designed to identify factors that trigger participants’ sexual-risk behaviors. This activity also provided information that allowed the facilitator to tailor the intervention to the unique risk and other characteristics (e.g., triggers) of each man by focusing on the particular risk behaviors and contexts most relevant to him. The intervention then focused on HIV/STI risk-reduction knowledge and perceived risk of HIV/STIs. A mini lecture covered HIV/STI symptoms, transmission, and prevention. A discussion of the participant’s goals and dreams and how his sexual-risk behaviors might affect his goals for himself, partners, and community introduced the take-home assignment, “Moving Towards My Goals,” which asked the participant to identify a behavioral goal based on his sexual risk identified earlier and to create a personal HIV sexual-risk-reduction plan he could employ to attain that goal.

In Session 2, activities addressed participants’ outcome expectancies regarding condom use and the correct and consistent use of condoms. It began with a review of the Session 1 take-home assignment, including participants’ barriers to achieving their personal behavioral goals and strategies to surmount the barriers, with facilitators following a different protocol depending on whether the participant had completed the assignment. A “Forced Choice” activity examined the participants’ beliefs about circumstances under which they should use condoms and the type of person who is vulnerable to HIV/STI. The facilitator demonstrated on an anatomical model correct condom use and then the participant practiced the correct steps for condom use on an anatomical model. The participants considered ways to make condom use fun and pleasurable and how alcohol and drug use might affect condom use. The facilitator also asked the participant to list excuses that he and his partners give for not wanting to use condoms and to think of responses to neutralize the excuses. A take-home assignment, “Creating a Relapse Prevention Plan,” asked the participant to imagine that he had been practicing safer sex and that he was suddenly faced with the triggers to have unsafe sex he had enumerated earlier. The facilitator asked the participant to identify ways he might avoid such triggers and to suggest discreet ways to have condoms available when needed.

In Session 3, the focus was on building knowledge, self-efficacy, and skills regarding negotiating condom use. A discussion centered on the Session 2 take-home assignment. The facilitator and participants enacted role-play scenarios about African American MSM negotiating condom use in an insertive or receptive role, with steady, casual, paying, or female partners, and under circumstances when they had slipped and had unsafe sex. Focus-group participants had liked the scenarios, saying they were realistic and employed relevant locations, including “Gay Acres,” where some men in the Philadelphia area meet other men for sex. The facilitator could adapt each role-play scenario to suit the information the participant previously provided to ensure that the scenarios was as authentic as possible. Participants learned and practiced negotiation and communication skills using “Say No, Explain Why, Provide Alternatives and Talk it Out (SWAT),” a four-step strategy to communicate effectively their decision to use condoms and abstain from unprotected intercourse. A “Virtual Sex Project” interactive video allowed participants to select personally relevant risk situations for actors in “hook-up” scenarios, scenarios that focus group participants said were realistic. The participants reviewed their personal HIV sexual-risk-reduction plan developed in Session 1 and explored ways to overcome potential obstacles and to sustain behavior change. In the “Commitment to Safety” activity, the participants wrote a safer-sex promise letter to self, partners, and community that was mailed to them 6 weeks after the intervention to remind them of their commitment to be safe sexually.

The time- and dosage-matched health-promotion intervention provided a control for “Hawthorne effects,” reducing the likelihood that the HIV/STI risk-reduction intervention’s effects could be attributed to non-specific features, including special attention [39]. Its activities, while similar to those in the HIV/STI risk-reduction intervention, focused on increasing physical activity and fruit-and-vegetable consumption and decreasing fat consumption to reduce the risk of chronic diseases, including heart disease, hypertension, stroke, diabetes, and certain cancers—leading causes of morbidity and mortality among African Americans [40].

We considered employing African American MSM as facilitators, but the focus groups with representatives of CBOs revealed that CBO staff who worked with African American MSM were mainly African American or Latino women of no particular sexual orientation self-identification and the African American MSM focus groups revealed no consensus on the desirable gender or sexual orientation of facilitators. Accordingly, we employed facilitators irrespective of gender and sexual orientation. The facilitators were 23 adults (17 women and 6 men) 28–64 years of age (mean age = 44.2). Twenty were African American, two were Latino, and one was white. About 56.5 % had a Master’s degree, which was the modal and median education; 79.1 % had previously facilitated HIV/STI risk-reduction interventions, 50.0 % had previously facilitated health-promotion interventions, and 65.2 % had previously worked with African American MSM. We hired individuals who had the basic skills to implement either of the two interventions. After stratifying them by gender and age, we randomly assigned them to be trained to implement one of the two interventions. In this way, we randomized facilitators’ characteristics across interventions; hence, reducing the plausibility of attributing any effects of the interventions to the facilitators’ pre-existing characteristics [24].

The facilitators received three 8-hour days of training in their assigned intervention, training that included a project overview, the theoretical framework, background information relevant to the assigned intervention, and effective facilitation techniques, including presentation style, time management, and nonverbal and verbal communication, and stressed the importance of implementation fidelity. The HIV/STI-risk-reduction-intervention training also covered transgender issues, sexual identity development, and “tops” versus “bottoms” (sexual positions). During the training, the trainers modeled the intervention activities, the facilitators learned their assigned intervention, practiced implementing it, received feedback from each other, the trainers, and investigators, and created common responses to potential issues that might arise during implementation. Besides the facilitator training, we provided facilitators and all staff who might have even incidental contact with the participants two 8-hour days of sensitivity training on the knowledge, skills, and perspectives necessary to work effectively with African American MSM.

We employed several quality assurance procedures. A facilitator supervisor met with the facilitator before each session, reviewing the materials, answering any questions, reviewing the session’s purpose, and reiterating any specific points that the facilitator should emphasize in the session. When the session ended, the supervisor reviewed the facilitator’s log sheets indicating the extent to which the facilitator completed the activities and debriefed him or her, addressing concerns and providing suggestions to address implementation issues. The supervisor also reviewed the digital tape recordings of the sessions and subsequently provided performance feedback to the facilitators and retraining if necessary. Periodically, the supervisor held group meetings with all the facilitators to discuss implementation issues and to fashion common responses.

### Assessments

The participants completed confidential questionnaires via audio computer-assisted self-interviewing (ACASI) technology, which provided both audio and video presentation of the questions and response options on a laptop computer. Sexual-risk behaviors, theoretical constructs, sociodemographic variables, and health-promotion behaviors and theoretical constructs were assessed pre-intervention and 6 and 12 months post-intervention. The theoretical constructs were also assessed immediately post-intervention. We pilot tested the paper version of the questionnaire with 217 men to ensure that the questions were clear and appropriate for the target population and then programmed it for ACASI and pilot tested it with 16 men to identify and correct any programming errors.

The primary outcome was consistent condom use, a binary variable reflecting whether the participant reported using a condom every time he had anal or vaginal intercourse in the past 90 days. It was based on a comparison of the sum of the reported anal and vaginal intercourse acts in the past 90 days and the sum of the reported condom-protected anal and vaginal intercourse acts in the past 90 days. Men who reported at least one intercourse act and whose number of reported protected acts equaled their number of acts were coded as practicing consistent condom use. Men who reported at least one intercourse act and whose reported number of protected acts was less than their number of acts were coded as not practicing consistent condom use. A widely used measure in HIV prevention trials [41], considerable evidence indicates that self-reported consistent condom use is associated with a reduced risk of STI, including HIV [42–46].

Secondary outcomes included proportion of condom-protected intercourse acts, unprotected sexual intercourse, multiple sexual partners, insertive anal intercourse, and receptive anal intercourse. The proportion of condom-protected intercourse acts was assessed in men who reported at least one intercourse act. The denominator was the sum of reported anal and vaginal intercourse acts in the past 90 days and the numerator was the sum of condom-protected anal and vaginal intercourse act in the past 90 days. Unprotected intercourse was a binary variable indicating whether the participants reported having vaginal or anal intercourse in the past 90 days without using a condom. It was constructed by subtracting the sum of the condom-protected anal and vaginal intercourse acts from the total number of anal and vaginal intercourse acts in the past 90 days. If the difference was one or greater the participant was coded as having unprotected intercourse; if the difference was zero or if the person reported no vaginal or anal intercourse in the past 90 days, the person was coded as not having unprotected intercourse. Participants whose sum of anal and vaginal intercourse partners in the past 90 days was 2 or greater were coded as having multiple partners, and those who reported having 0 or 1 anal and vaginal intercourse partners in the past 90 days were coded as not having multiple partners. Insertive anal intercourse was a binary variable indicating whether the participant reported having insertive anal intercourse with a man in the past 90 days. Receptive anal intercourse was a binary variable indicating whether the participant reported having receptive anal intercourse with a man in the past 90 days.

#### Outcome Expectancies

Table 1 presents the number of items, response format, and Cronbach’s alpha for the theoretical constructs. We assessed three types of outcome expectancies regarding condom use. Hedonistic outcome expectancy concerns the belief that the use of condoms will not interfere with sexual enjoyment [9, 11, 23, 24, 34–37, 47, 48]. It was measured with a scale used in previous intervention trials [11, 37] to which two items were added based on qualitative research. The new scale correlates highly with the original scale, *r* (593) = 0.98, p < 0.0001. An example item is “When a condom is used, sex is more fun.” Prevention outcome expectancy, the belief that condoms can reduce the risk of HIV, other STI, and pregnancy, was assessed with a scale used in previous research [24, 34, 36, 38, 48]. An example item is “Condoms help prevent AIDS.” Self-evaluative outcome expectancy, the expected reactions of pride as a consequence of using condoms [9, 23, 35], was measured with a scale used in previous research [49]. An example item is “I feel good about myself when I use condoms.”

**Table 1:** Characteristics of theoretical constructs

Construct	Number of items	Type of response	Alpha
Condom-use hedonistic outcome expectancy	9	5-point Likert	0.87
Condom-use prevention outcome expectancy	3	5-point Likert	0.92
Condom-use self-evaluative outcome expectancy	3	5-point Likert	0.70
Condom availability self-efficacy	5	5-point Likert	0.68
Condom-use negotiation self-efficacy	5	5-point Likert	0.75
Condom-use technical skill self-efficacy	11	5-point Likert	0.93
Condom-use impulse-control self-efficacy	3	5-point Likert	0.87
HIV risk-reduction knowledge	16	True/False	–
Condom-use knowledge	5	True/False	–
Condom-use subjective norm	5	5-point Likert	0.91
Condom-use descriptive norm	3	5-point Likert	0.83

Ratings on the Likert scales could range from 1 (Disagree strongly) to 5 (Agree strongly) except for condom-use descriptive norm where the ratings could range from 1 (never) to 5 (every time). The score was the mean of the ratings except for HIV risk-reduction knowledge and condom use knowledge where the score was the sum of the number of items correctly answered. Alpha is Cronbach’s coefficient alpha for the post-intervention assessment of the construct, which was analyzed as the potential mediator

#### Self-efficacy

We assessed four types of self-efficacy regarding condom use. Availability self-efficacy, the man’s belief that he can have condoms available when needed [11, 24], was assessed with a scale used in previous research [24]. An example item is “It is easy for me to have a condom with me all the time.” Negotiation self-efficacy, the man’s belief that he can convince his partners to use condoms [8, 24, 37], was assessed with a scale used in previous research [24, 37] to which two items were added. The new scale correlates highly with the original scale, *r* (593) = 0.95, p < 0.0001. An example item is “I can get my partner to use a condom, even if he or she doesn’t want to.” Technical skill self-efficacy, the man’s belief that he knows how to use condoms [8, 24, 36, 37], was assessed with a scale that predicted intention to use condoms in the pilot survey of African American MSM, *r* (203) = 0.43, p < 0.0001. An example item is “I can use a condom, even if the room is dark.” Impulse-control self-efficacy, the man’s belief that he can control himself sufficiently when sexually aroused to use a condom [11, 24, 37], was measured with a scale used in previous research [24, 37]. An example item is “If I am sexually aroused, I can stop before sex to use a condom.”

HIV/STI risk-reduction knowledge regarding transmission of HIV, risk of different behaviors, and correct use of condoms was assessed with a modified version of an index used in previous research [37]. One item on limiting partners was added. Condom-use knowledge is a subscale of the HIV/STI risk-reduction knowledge index consisting of items on the correct use of condoms [37].

We also assessed two theoretical constructs that, though not targeted by the intervention, are constructs in our theoretical framework [15, 50]. Subjective norm is the man’s belief regarding whether people important to him would approve of his using condoms [51]. An example item is “Most people who are important to me would think it is okay for me to use a condom.” Condom-use descriptive norm is the man’s belief regarding his closest friends’ frequency of using condoms [51]. An example item is “On average, how often do your 5 closest friends use condoms when they have sexual intercourse?”

Data collectors received two 8-hour days of training that included modeling of data-collection procedures and practice with performance feedback. We employed procedures used in previous trials to increase the validity of self-reported sexual behavior [52, 53]. For instance, to facilitate participants’ recall, we asked them to report their behaviors during a brief period (i.e., past 90 days), posted the dates comprising the period on newsprint, gave them calendars highlighting the period, and instructed them to record some events that occurred during the period. To reduce the likelihood that participants would minimize or exaggerate, we utilized ACASI, which has been shown to increase reports of socially undesirable behaviors as compared with face-to-face interviews and pencil-and-paper surveys, which may reflect more accurate responding [54, 55]. In addition, we stressed the importance of responding honestly, informing them that their responses would be used to create programs for African American MSM like themselves and that we could do so only if they answered the questions honestly. We assured the participants that their responses would be kept confidential [56] and that code numbers rather than names would be used on the questionnaires. Participants signed an agreement pledging to answer the questions honestly, a procedure that has been shown to yield more truthful self-reports [57].

### Sample size and Statistical Analysis

A statistical power analysis was performed to calculate the sample size required to detect a clinically significant effect of the HIV/STI risk-reduction intervention on the primary outcome, consistent condom use, compared with the attention-control group. In the pilot survey, we found that 42 % of the African American MSM reported consistent condom use over all of their anal and vaginal intercourse acts in the past 90 days. We selected an absolute increase of 14 % points in consistent condom use as a clinically and substantively important effect size. Assuming a two-tailed test, α = 0.05, 20 % attrition, and a 14 % increase in consistent condom use from 42 % in the control group to 56 % in the HIV/STI intervention group, with N = 594 men enrolled in the trial, the estimated statistical power was 84 % [58].

We used descriptive statistics to summarize the participants at baseline on socio-demographic variables and χ^2^ test and logistic regression to analyze attrition. The efficacy of the HIV/STI risk-reduction intervention averaged over the 6- and 12-month follow-ups compared with the health-promotion intervention was tested using logistic generalized-estimating-equations (GEE), adjusting for the longitudinal repeated measurements on participants [59, 60] and controlling for baseline measure of the outcome. The models were fit and contrast statements specified to obtain estimated odds ratios and their corresponding 95 % confidence intervals (CI). Robust standard errors were used and an independent working correlation matrix was specified.

The models included time-independent covariates, baseline measure of the outcome, intervention condition, and time (two categories representing 6- and 12-month follow-up). In addition, we included as covariates sexual orientation self-identification, self-reported HIV status, and age group in analyses in which they were statistically significant. We report estimated intervention standardized effect sizes (ds) averaged over the two follow-up assessments, calculated by transforming the odds ratios using the Cox transformation [61]. Models assessing whether the efficacy of the intervention differed between the two follow-ups included the baseline measure of the criterion, intervention condition, time, and the Intervention-Condition × Time interaction. The analyses were performed using an intent-to-treat model with participants analyzed based on their intervention assignment, regardless of the number of intervention or data-collection sessions they attended. Analyses were completed using SAS V9.

We assessed mediation using a product-of-coefficients approach [7, 62], where the α path denotes the effect of the intervention on a potential mediator at the immediate post-intervention assessment, the β path denotes the effect of the potential mediator on consistent condom use averaged over the 6- and 12-month post-intervention assessments, and the product of α and β (αβ) quantifies the mediated effect of the intervention. Mediation is determined by testing whether the αβ product differs significantly from ‘0’. Each theoretical construct was evaluated separately for mediation of effects of the intervention on the primary outcome, consistent condom use. We estimated the α paths using linear regression models on theoretical constructs at the immediate post-intervention assessment, adjusting for baseline of the theoretical construct and consistent condom use. We estimated the β paths using GEE logistic regression models with time, intervention condition, and baseline of the theoretical construct and consistent condom use as covariates. Estimated mean differences and 95 % CI are reported for the α paths. Estimated odds ratios and 95 % CI are reported for the β paths. Estimated αβ products and, because the distribution of a product is non-normal, asymmetric 95 % confidence intervals (ACI) calculated using the bootstrap quantile method [62] with 2,000 replicates are reported. The p < 0.05, two-tailed statistical significance criterion was used. Mediation analyses were conducted in R version 2.15.1 [63].

## Results

### Characteristics of the Sample

Table 2 presents characteristics of participants by condition. The participants were 595 African American MSM: 295 in the HIV/STI risk-reduction intervention and 300 in the health-promotion control intervention. Participants’ age ranged from 18 to 69 years (mean = 41.6; SD = 10.7). Only 28.5 % were employed, and 48.4 % had completed high school. Almost all had been tested for HIV, and of those tested, 29.5 % said they were HIV positive. There were several indicators of high risk: 48.9 % had a history of childhood sexual abuse victimization, 37.1 % had a history of intimate partner violence victimization, 44.5 % were alcohol dependent, and 51.8 % had a history of incarceration. A large minority reported substance use in the past 90 days, including marijuana (39.0 %) and crack cocaine (18.9 %). About 43.7 % said they had intercourse with a woman in the past 90 days. About 40.6 % self-identified as gay, 41.3 % self-identified as bisexual, 10.5 % said they were on the down low, and 7.6 % self-identified as straight.

**Table 2:** Baseline sociodemographic characteristics of African American MSM by intervention condition, Philadelphia, PA, 2008–2011

Characteristic	Total no. (%) or Mean (SE)	Health intervention no. (%) or Mean (SE)	HIV/STI intervention no. (%) or Mean (SE)
Age (years)	41.64 (0.44)	41.85 (0.61)	41.44 (0.63)
Completed high school	287/593 (48.4)	137/298 (46.0)	150/295 (50.8)
Unemployed	424/593 (71.5)	218/298 (73.2)	206/295 (69.8)
Monthly income			
Less than $400	219/593 (36.9)	112/298 (37.6)	107/295 (36.3)
$400 to $850	212/593 (35.8)	98/298 (32.9)	114/295 (38.6)
$851 to $1,650	119/593 (20.1)	67/298 (22.5)	52/295 (17.6)
More than $1,650	43/593 (7.3)	21/298 (7.0)	22/295 (7.5)
Stable housing	463/593 (78.1)	233/298 (78.2)	230/295 (78.0)
Married	38/593 (6.4)	21/298 (7.0)	17/295 (5.8)
Sexual self-identity			
Gay	241/593 (40.6)	113/298 (37.9)	128/295 (43.4)
Straight	45/593 (7.6)	25/298 (8.4)	20/295 (6.8)
Bisexual	245/593 (41.3)	121/298 (40.6)	124/295 (42.0)
On the down low	62/593 (10.5)	39/298 (13.1)	23/295 (7.8)
Intercourse with a woman in the past 90 days	259/593 (43.7)	130/298 (43.6)	129/295 (43.7)
Ever tested for HIV	568/593 (96.0)	284/298 (95.3)	284/295 (96.3)
HIV positive	168/569 (29.5)	85/285 (29.8)	83/284 (29.2)
Sexual abused as a child	290/593 (48.9)	147/298 (49.3)	143/295 (48.5)
Intimate partner violence victim	220/593 (37.1)	116/298 (38.9)	104/295 (35.2)
Alcohol dependent^a^	264/593 (44.5)	121/298 (40.6)	143/295 (48.5)
Drug dependent^b^	99/593 (16.7)	47/298 (15.8)	52/295 (17.6)
Ever incarcerated	307/593 (51.8)	159/298 (53.4)	148/295 (50.2)

Stable housing was coded “1” for men living in their own, their family’s or someone else’s home or apartment and “0” for men living in a rooming house or single room hotel, welfare type place, or group home or institution and for those with no regular place to live

*MSM* men who have sex with men

^a^Based on a score of 2 or greater on the CAGE (Cutting down, Annoyance by criticism, Guilty feeling, and Eye-openers) questionnaire

^b^Based on a score of 3 or greater on the TCUDS (Texas Christian University Drug Screen) questionnaire

**Fig. 1:**
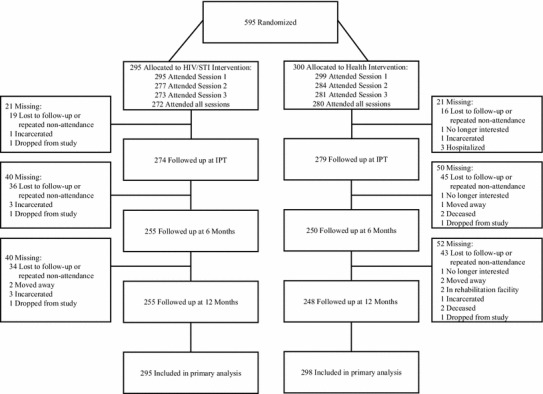
Progress of participating African American men who have sex with men through the trial, Philadelphia, PA, 2008–2012

As shown in Fig. 1, attendance at the 3 intervention sessions was excellent: 594 or 99.8 % attended Intervention Session 1; 561 or 94.3 % attended Intervention Session 2; and 554 or 93.1 % attended Intervention Session 3. A high percentage of participants reported completing take-home assignment 1 (483/554 or 87.2 %) and 2 (500/554 or 90.3 %), with a higher percentage of HIV/STI risk-reduction (251/273 or 91.9 %) compared with control (232/281 or 82.6 %) participants reporting completing assignment 1 (p = 0.001). On average, the facilitators reported completing 98.0 % (SD = 5.6 %) of the intervention activities. High percentages of participants completed the post-intervention assessments: 553 or 92.9 % completed the immediate post-test; 505 or 84.9 % completed the 6 months post-intervention follow-up; 503 or 84.5 % completed the 12 months post intervention follow-up. Of the original 595, 538 or 90.4 % attended at least one of the two follow-ups. The HIV/STI risk-reduction (91.2 %) and control (89.7 %) conditions did not differ significantly in the percentage attending at least one follow-up (p = 0.5288).

Baseline measures of outcomes did not predict attending at least one follow-up, nor did facilitators’ sex, age, or experience working with African American MSM. In addition, none of the baseline sociodemographic characteristics predicted returning for at least one follow-up, with three exceptions: Age was positively associated with returning for follow-up: The older the participants were, the more likely they were to return (p < 0.004). Men who had stable housing (92.2 %) were more likely to return (p = 0.009) than were those who had unstable housing (84.6 %). Among men who reported being tested for HIV, those who said they were HIV positive (98.2 %) were more likely to return (p < 0.0001) than were men who said they were HIV negative (87.3 %).

### Effects of the BRO HIV/STI Risk-Reduction Intervention on Sexual Behaviors

Table 3 presents the descriptive statistics for sexual behavior outcomes by intervention condition and assessment period. Table 4 presents estimated intervention effects unadjusted and adjusted for baseline response and other significant covariates. Irrespective of condition, self-reported consistent condom use in the past 90 days increased significantly averaged over the 6- and 12-month follow-up compared with baseline (p < 0.0001). The HIV/STI risk-reduction and health-promotion interventions did not differ significantly on consistent condom use averaged over the 6- and 12-month follow-up assessments, adjusting for baseline consistent condom use.

**Table 3:** Self-reported sexual behaviors by intervention condition and assessment period, African American MSM, Philadelphia, PA 2008–2012

Self-reported sexual behavior	Baseline		6-month		12-month	
	Health intervention no (%) or Mean (SE)	HIV/STI intervention no (%) or Mean (SE)	Health intervention no (%) or Mean (SE)	HIV/STI intervention no (%) or Mean (SE)	Health intervention no (%) or Mean (SE)	HIV/STI intervention no (%) or Mean (SE)
Consistent condom use	142/273 (52.0)	147/275 (53.4)	124/190 (65.3)	128/203 (63.0)	112/190 (59.0)	124/194 (63.9)
Proportion condom-protected intercourse	0.723 (0.023)	0.749 (0.021)	0.770 (0.027)	0.766 (0.026)	0.722 (0.029)	0.767 (0.027)
Unprotected intercourse	125/273 (45.8)	122/275 (44.4)	66/187 (35.3)	74/202 (36.6)	76/188 (40.4)	69/193 (35.8)
Multiple sexual partners	230/285 (80.7)	248/284 (87.3)	121/239 (50.6)	139/244 (57.0)	122/236 (51.7)	119/245 (48.6)
Insertive anal intercourse	225/285 (79.0)	219/284 (77.1)	109/239 (45.6)	122/244 (50.0)	96/236 (40.7)	101/245 (41.2)
Receptive anal intercourse	125/285 (43.9)	134/284 (47.2)	72/239 (30.1)	68/244 (27.9)	76/236 (32.2)	62/245 (25.3)

*MSM* men who have sex with men

**Table 4:** GEE empirical significance tests, Odds Ratios (OR), and 95 % confidence intervals (CI) for the overall intervention effect unadjusted and adjusted for baseline prevalence and significant covariates, African American MSM, Philadelphia, PA 2008–2012

Outcome	Unadjusted			Adjusted for baseline		
	OR (95 % CI)	p value	d	OR (95 % CI)	p value	d
Consistent condom use	1.03 (0.73, 1.45)	0.8658	0.02	1.01 (0.71, 1.44)	0.9504	0.01
Proportion condom-protected intercourse	1.55 (0.87, 2.77)	0.1362	0.27	1.58 (0.89, 2.84)	0.1210	0.28
Unprotected intercourse	0.96 (0.68, 1.35)	0.8098	−0.03	0.99 (0.69, 1.42)	0.9612	0.00
Multiple sexual partners	1.07 (0.80, 1.43)	0.6641	0.04	0.96 (0.71, 1.30)	0.7949	−0.02
Insertive anal intercourse	1.11 (0.81, 1.51)	0.5244	0.06	1.12 (0.81, 1.54)	0.4982	0.05
Receptive anal intercourse	0.80 (0.57, 1.13)	0.2016	−0.14	0.64 (0.44, 0.94)	0.0218	−0.27

The intervention effect is averaged over the 6-month and 12-month post-intervention assessments. All adjusted analyses adjust for baseline of the criterion. Proportion of condom-protected, insertive anal intercourse, and receptive anal intercourse also adjusted for sexual self-identification. Multiple partners also adjusted for self-reported HIV status. Insertive anal intercourse also adjusted for age group. d is the effect size estimate in standard deviation units based on Cox transformation of the odds ratio [61]

*GEE* generalized estimating equations, *MSM* men who have sex with men

Irrespective of condition, participants were less likely to report unprotected intercourse (p < 0.0001), multiple partners (p < 0.0001), insertive anal intercourse (p < 0.0001), and receptive anal intercourse (p < 0.0001) averaged over the 6- and 12-month follow-ups compared with baseline. Men in the HIV/STI risk-reduction intervention were less likely to report having receptive anal intercourse during the follow-up period than were their counterparts in the health-promotion intervention, adjusting for baseline consistent condom use and sexual orientation self-identification. The HIV/STI risk-reduction and health-promotion interventions did not differ significantly on proportion of condom-protected intercourse acts, unprotected sexual intercourse, multiple partners, or insertive anal intercourse during the follow-up period. The Intervention x Follow-up interactions were nonsignificant, indicating that efficacy of the intervention did not differ significantly at 6-month compared with 12-month follow-up for any outcome. In addition, the intervention’s efficacy did not differ by the participants’ HIV status or the facilitator’s sex, age, or experience working with African American MSM.

### Mediation Analysis of the Intervention Effect on Consistent Condom Use

The means and standard errors for the theoretical constructs by intervention condition and assessment period are presented in Table 5. The results of the mediation analysis are presented in Table 6. Compared with the health-control intervention, BRO significantly increased seven of the nine theoretical constructs it targeted, adjusting for baseline of the theoretical construct and consistent condom use: condom-use hedonistic outcome expectancy, prevention outcome expectancy, self-evaluative outcome expectancy, technical-skill self-efficacy, and impulse-control self-efficacy, HIV/STI risk-reduction knowledge, and condom-use knowledge. It did not significantly increase condom-use availability or condom-use negotiation self-efficacy.

**Table 5:** Mean and ± Standard Error for theoretical constructs by intervention condition and assessment period, African American MSM, Philadelphia, PA 2008–2012

Theoretical construct	Baseline		Post-intervention		6 months		12 months	
	Health intervention N = 298	HIV/STI intervention N = 295	Health intervention N = 281	HIV/STI intervention N = 273	Health intervention N = 251	HIV/STI intervention N = 254	Health intervention N = 241	HIV/STI intervention N = 254
Targeted by the HIV/STI intervention								
Condom-use hedonistic outcome expectancy	3.5 ± 0.05	3.52 ± 0.04	3.7 ± 0.05	3.92 ± 0.04	3.62 ± 0.05	3.8 ± 0.04	3.7 ± 0.04	3.78 ± 0.05
Condom-use prevention outcome expectancy	4.42 ± 0.04	4.48 ± 0.04	4.4 ± 0.05	4.58 ± 0.04	4.35 ± 0.05	4.47 ± 0.04	4.29 ± 0.06	4.39 ± 0.05
Condom-use self-evaluative outcome expectancy	4.22 ± 0.04	4.16 ± 0.04	4.27 ± 0.04	4.4 ± 0.04	4.23 ± 0.04	4.3 ± 0.04	4.13 ± 0.05	4.25 ± 0.05
Condom-use availability self-efficacy	4.29 ± 0.03	4.31 ± 0.03	4.26 ± 0.04	4.36 ± 0.04	4.26 ± 0.04	4.31 ± 0.04	4.26 ± 0.04	4.23 ± 0.04
Condom-use negotiation self-efficacy	4.03 ± 0.04	4.02 ± 0.04	4.07 ± 0.04	4.14 ± 0.04	4.03 ± 0.04	4.12 ± 0.04	4 ± 0.05	4.02 ± 0.05
Condom-use technical skills self-efficacy	4.07 ± 0.04	4.03 ± 0.04	4.14 ± 0.04	4.25 ± 0.04	4.2 ± 0.04	4.19 ± 0.04	4.16 ± 0.05	4.17 ± 0.05
Condom-use impulse-control self-efficacy	3.77 ± 0.06	3.66 ± 0.06	3.95 ± 0.05	4.05 ± 0.05	3.88 ± 0.06	4.01 ± 0.06	3.9 ± 0.06	3.95 ± 0.06
HIV risk-reduction knowledge	12.13 ± 0.16	12.23 ± 0.14	12.6 ± 0.16	13.83 ± 0.14	12.65 ± 0.17	13.04 ± 0.16	12.5 ± 0.19	12.93 ± 0.18
Condom-use knowledge	4.31 ± 0.06	4.33 ± 0.05	4.37 ± 0.06	4.59 ± 0.05	4.48 ± 0.06	4.56 ± 0.05	4.41 ± 0.07	4.5 ± 0.06
Not targeted by the HIV/STI intervention								
Condom-use subjective norm	4.4 ± 0.04	4.34 ± 0.05	4.44 ± 0.04	4.46 ± 0.04	4.34 ± 0.04	4.37 ± 0.05	4.34 ± 0.05	4.41 ± 0.04
Condom-use descriptive norm	2.74 ± 0.05	2.67 ± 0.05	2.69 ± 0.06	2.73 ± 0.06	2.79 ± 0.06	2.84 ± 0.06	2.62 ± 0.06	2.86 ± 0.06

**Table 6:** GEE mediation analysis of intervention effects (HIV/STI intervention Vs health promotion intervention) fit to consistent (100 %) condom use 6 and 12 months post-intervention, African American Men, Philadelphia, PA 2008–2012

Theoretical construct	Alpha path		Beta path		Indirect effect
	Mean difference (95 % CI)	p value	Odds Ratio (95 % CI)	p value	Alpha–Beta product (95 % ACI)
Targeted by the HIV/STI intervention					
Condom-use hedonistic outcome expectancy	0.19 (0.09, 0.29)	<0.001	1.16 (0.83, 1.63)	0.388	0.03 (−0.04, 0.10)
Condom-use prevention outcome expectancy	0.15 (0.04, 0.26)	0.006	0.80 (0.62, 1.04)	0.095	−0.03 (−0.08, 0.00)
Condom-use self-evaluative outcome expectancy	0.14 (0.04, 0.24)	0.007	1.33 (0.99, 1.78)	0.057	0.04 (−0.00, 0.10)
Condom-use availability self-efficacy	0.08 (−0.01, 0.17)	0.076	0.83 (0.60, 1.17)	0.286	−0.01 (−0.06, 0.01)
Condom-use negotiation self-efficacy	0.05 (−0.04, 0.15)	0.281	1.47 (1.07, 2.02)	0.017	0.02 (−0.01, 0.08)
Condom-use technical skills self-efficacy	0.15 (0.05, 0.25)	0.004	0.94 (0.71, 1.26)	0.690	−0.01 (−0.06, 0.04)
Condom-use impulse-control self-efficacy	0.15 (0.02, 0.28)	0.020	1.34 (1.07, 1.69)	0.011	0.04 (0.00, 0.11)
HIV risk-reduction knowledge	1.17 (0.81, 1.54)	<0.001	0.99 (0.91, 1.07)	0.773	−0.01 (−0.12, 0.09)
Condom-use knowledge	0.19 (0.05, 0.34)	0.009	0.90 (0.73, 1.10)	0.302	−0.02 (−0.09, 0.00)
Not targeted by the HIV/STI intervention					
Condom-use subjective norm	0.04 (−0.06, 0.13)	0.456	1.22 (0.91, 1.64)	0.189	0.01 (−0.01, 0.04)
Condom-use descriptive norm	0.04 (−0.11, 0.18)	0.620	1.46 (1.15, 1.85)	0.002	0.01 (−0.04, 0.08)

Theoretical constructs are from the immediate post-intervention assessment. Alpha path, the effect of the intervention on the theoretical construct, is adjusted for baseline consistent condom use and the theoretical construct. Beta path, the relation of the theoretical construct to consistent condom use 6 and 12 months post-intervention, is adjusted for intervention and baseline of consistent condom use and mediator. CI is confidence interval. ACI is asymmetric confidence interval based on bootstrap quantile method with 2,000 replicates

The β path was significant for two of the nine theoretical constructs the intervention targeted, condom-use negotiation self-efficacy and condom-use impulse-control self-efficacy, and one construct the intervention did not target, condom-use descriptive norm. Only one theoretical construct the intervention targeted, condom-use impulse-control self-efficacy, had a significant αβ product indicating BRO had an indirect effect, increasing consistent condom use through increased condom-use impulse-control self-efficacy.

## Discussion

Contrary to expectation, the present study did not support the hypothesis that the HIV risk-reduction intervention would increase consistent condom use in African American MSM compared with the attention-matched control group. Although consistent condom use did increase significantly in the sample as a whole, the increase was not greater in the HIV risk-reduction intervention. The HIV risk-reduction intervention reduced receptive anal intercourse compared with the control group, a behavior tied to elevated risk of incident HIV infection [64], but did not increase the proportion of condom-protected intercourse or decrease multiple partners or insertive anal intercourse.

In finding limited intervention effects on sexual-risk behavior, the present study is similar to other studies on African American MSM. For instance, the earliest trial to test an intervention with African American MSM found no difference in sexual behavior between the intervention and a no-treatment control group at 12- or 18-month follow-up [13]. Two more recent studies found no difference in sexual behavior between intervention and control groups at 3-month follow-up [4, 5]. Although a trial found that the Many Men, Many Voices intervention reduced one sexual-risk behavior, unprotected insertive intercourse with causal partners, averaged over 3- and 6-month post-intervention follow-ups, the intervention did not reduce unprotected anal intercourse with main or causal partners, receptive intercourse with main or causal partners, unprotected insertive anal intercourse with main partners, or the number of partners compared with the wait-list control group [3].

Consistent with several other trials, we also found sexual-risk behaviors decreased over time in the sample as a whole [4, 9, 25]. Consistent condom use increased and multiple partners, insertive anal intercourse, and receptive anal intercourse decreased over time. We would speculate about a couple factors that may account for the overall risk reduction. First, men who agreed to participate in the study may have been interested in reducing their sexual-risk behaviors, which both prompted their decision to participate and subsequently to reduce their risk behaviors in the post-intervention period. Second, the repeated completion of the risk-behavior assessments may have constituted an intervention that prompted the men to think about their behavior and subsequently act to reduce their sexual risks [9].

While there have been calls for mediation analyses of HIV risk-reduction interventions [65], we are unaware of any other trial with African American MSM that has reported a mediation analysis. Indeed, most HIV risk-reduction intervention trials targeting African American MSM have not reported whether the intervention affected theoretical constructs hypothesized to account for the mechanism of its impact [3, 5, 13]. One trial found that the intervention did not change social-cognitive-theory constructs compared with the control group [4].

The present mediation analysis provided insight into why the intervention did not affect the primary outcome of consistent condom use. Generally, mediation analysis provides information on two sets of relationships: which potential mediators were changed by the intervention; and which potential mediators were associated with changes in the outcome. In the present analysis, the intervention changed seven potential mediators, constructs from social cognitive theory and the reasoned action approach the intervention targeted. However, of these, only one, condom-use impulse-control self-efficacy, was related to consistent condom use and, consequently, was the only significant mediator. On the other hand, the intervention did not increase condom-use negotiation self-efficacy, which was significantly related to consistent condom use. Given that neither the participants’ significant referents nor their closest friends attended the intervention, it is not surprising that the intervention also did not affect the two norms-related constructs, subjective norms and descriptive norms, though one of them, descriptive norms, predicted increased consistent condom use.

The intervention increased hedonistic outcome expectancy, self-evaluative outcome expectancy, and condom-use technical skills self-efficacy, and previous research has tied these constructs to condom use or condom-use intention in a variety of populations, including MSM [9, 35, 66], African American adolescents [36], South African adolescents [67], college students in South Africa [68], college students in the U.S. [69], and Flemish high school students [70]. For instance, a study found that hedonistic outcome expectancy and self-evaluative outcome expectancy predicted sexual-risk behavior following an intervention with MSM [9]. What is puzzling is why condom-use hedonistic outcome expectancy, self-evaluative outcome expectancy, and technical skills self-efficacy did not predict consistent condom use in the present trial.

The results of the mediation analysis have implications for developing efficacious interventions for African American MSM. Enhancing the existing skill-building activities or adding additional activities to bolster self-efficacy to negotiate condom use might increase BRO’s efficacy. Another implication is that enhancing effects on descriptive norms might increase the efficacy of interventions for African American MSM more generally. To be sure, pursuing increases in descriptive norms would require a different intervention strategy: one-on-one interventions or interventions with groups of strangers are unlikely to affect descriptive norms because there is little reason for participants to perceive that their closest friends’ condom use has changed since the friends have not received any intervention. More likely to affect descriptive norms is intervening not only with individual African American MSM, but also with their closest friends. By so doing, it may be possible to change the friends’ behaviors, which would affect the participants’ descriptive norm, which would, in turn, increase consistent condom use, particularly if the intervention also increased condom-use negotiation self-efficacy and impulse-control self-efficacy.

The limitations of this study should be considered. Behavior was measured with self-reports, which may be subject to social desirability bias. Although the use of ACASI may have mitigated potential problems with self-reports, objective indicators of sexual-risk behavior such as biologically confirmed STIs would have improved the study. In addition, the findings may not generalize to all African American MSM because participants were not randomly selected. The reliability of the theoretical constructs ranged from 0.68 to 0.93. Higher reliability would have increased the statistical power for the mediation analyses; hence, we may have underestimated mediation [71]. A limitation of the mediation analyses is that they are correlational; evidence from factorial experiments manipulating intervention components and putative mediators would be more cogent, though admittedly difficult to implement in practice [72].

There were also important strengths. Behavior-change theory was integrated with extensive formative research to develop an intervention that was both theoretically grounded and culturally congruent. A RCT design and a dose- and modality-equivalent control intervention, controlling special attention, was employed. The retention rate was relatively high and did not differ by intervention arm. Mediation analysis was used to suggest an alternative intervention approach.

## Conclusion

Given the paucity of efficacious HIV risk-reduction interventions for African American MSM, the population at highest risk for HIV in the US, this study contributes to the literature by suggesting new directions for intervention research with this population. Consistent with several other trials, we found scant evidence that the intervention reduced sexual-risk behavior. Although it reduced receptive anal intercourse, and meta-analytic evidence indicates MSM who engage in receptive anal intercourse only or both receptive and insertive anal intercourse are over six times more likely to develop an incident HIV infection compared with MSM who engage in insertive anal intercourse only [64], it did not increase consistent condom use, the primary outcome, or affect any other behavioral outcome. However, the trial, employing a high-risk sample that reported many syndemic psychosocial conditions, including childhood abuse victimization, intimate partner violence, and alcohol dependency [73], went beyond previous trials in drawing attention to the mediating mechanism in a theory-based intervention.

Descriptive norm, the man’s belief that his closest friends are using condoms, was a significant predictor of consistent condom use in the mediation analysis, a finding that raises the possibility that interventions designed to increase descriptive norms might be efficacious with African American MSM. Interventions such as the one that we employed, focusing exclusively on MSM and not their close friends, are unlikely to change MSM’s perceptions of their close friends’ condom use. An efficacious approach might be to incorporate MSM and their close friends in interventions with the goal of changing descriptive norms, which, in turn, would increase safer behavior, including consistent condom use. By conducting research with this alternative strategy, it may possible to reduce the high rates of new HIV infections in African American MSM.
